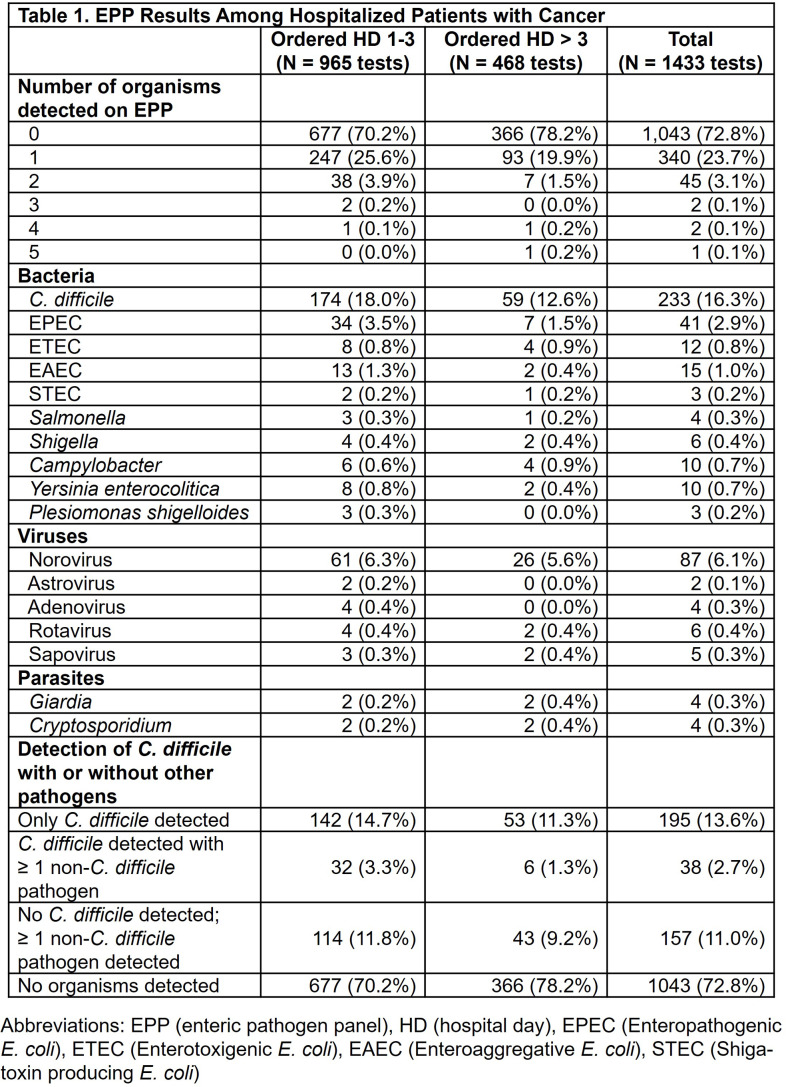# 26 Diagnostic Stewardship to Reduce Beta-D-Glucan Testing Using Clinical Decision Support

**DOI:** 10.1017/ash.2026.10471

**Published:** 2026-06-23

**Authors:** Emily Rosen, Shandra Truong, Elizabeth M. Krantz, Allison Thibodeau, Cynthia Wallace, Frank Tverdek, Zahra Kassamali Escobar, Pooja Bhattacharyya, Regina Lengermann, Michelle Swetky, Salma Walji, Marie Wilson, Lori Bourassa, Sandra Olson, Joanne Lang, Masumi Ueda Oshima, Steven Pergam, Catherine Liu

**Affiliations:** 1 Fred Hutch Cancer Center / University of Washington; 2 Vaccine and Infectious Disease Division, Fred Hutch Cancer Center; 3 Fred Hutch Cancer Center; 4 Fred Hutchinson Cancer Center; 5 University of Washington; 6 FHCC; 7 University of Washington Medical Center Montlake Campus

## Abstract

**Background:** Diarrhea is common among patients with cancer, and rapid diagnosis of gastrointestinal (GI) infections using a multiplex stool enteric pathogen panel (EPP) may expedite clinical decision-making and inform infection prevention measures. However, the diagnostic yield of EPPs in patients with cancer with hospital-onset diarrhea is not well defined. At our center, inpatient diarrhea testing guidelines recommend EPP testing primarily in those with community-onset diarrhea. We aimed to assess adherence to these EPP testing guidelines and describe EPP diagnostic yield in hospitalized patients with cancer to identify opportunities for diagnostic stewardship. **Methods:** We retrospectively reviewed EPP and stand-alone C. difficile (SACD) tests ordered between July 1, 2021 – May 23, 2025 among hospitalized patients (age ≥18 years) with a malignancy diagnosis. Our center recommends EPP testing (BIOFIRE® FILMARRAY® GI Panel) for community-onset diarrhea (on or before hospital day [HD] 3) and SACD testing for hospital-onset diarrhea (after HD 3). EPPs may be ordered after HD 3 for patients with unexplained prolonged or bloody diarrhea. **Results:** A total of 3338 stool tests (1433 EPP, 1905 SACD) were performed among 2131 patients. Among EPPs, 468 (33%) were ordered after HD 3 whereas 1621 (85%) SACD tests were ordered after HD 3 (Figure 1). The proportion of EPPs ordered after HD 3 was highest among patients with hematologic malignancies (44%) compared to other cancer types and higher among hematopoietic cell transplant (HCT) recipients versus non-HCT recipients (54% vs. 22%; Figure 2). Among EPPs ordered after HD 3, 102/468 (22%) were positive for ?1 pathogen compared to 288/965 (30%) EPPs ordered on HD 1-3. At least 1 non-C. difficile pathogen was detected in 49/468 (10%) EPPs ordered after HD 3 compared to 146/965 (15%) on HD 1-3 (Table 1). Among EPPs with non-C. difficile pathogens detected after HD 3, norovirus was most frequent (26/468; 6%; Table 1). **Conclusion:** EPP detection of non-C. difficile pathogens among hospitalized patients with cancer was lower in patients with hospital-onset diarrhea compared to those with community-onset diarrhea. Most EPP orders after HD 3 at our center occurred in HCT recipients and patients with hematologic malignancies. Additional studies are needed to determine the clinical significance of positive EPP results beyond HD 3 and understand which patients may benefit most from EPP testing beyond HD 3.

**Figure:**
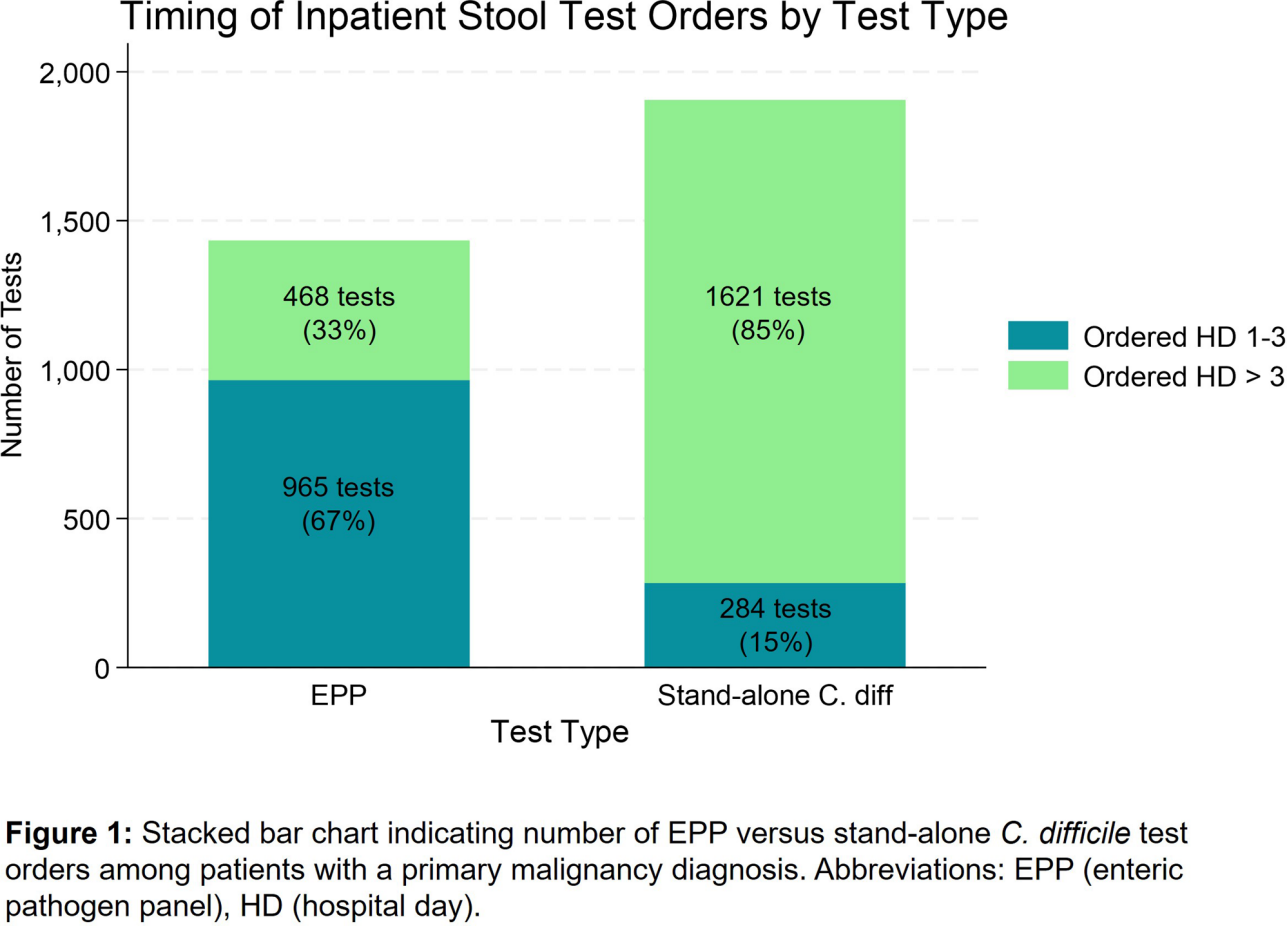

**Figure:**
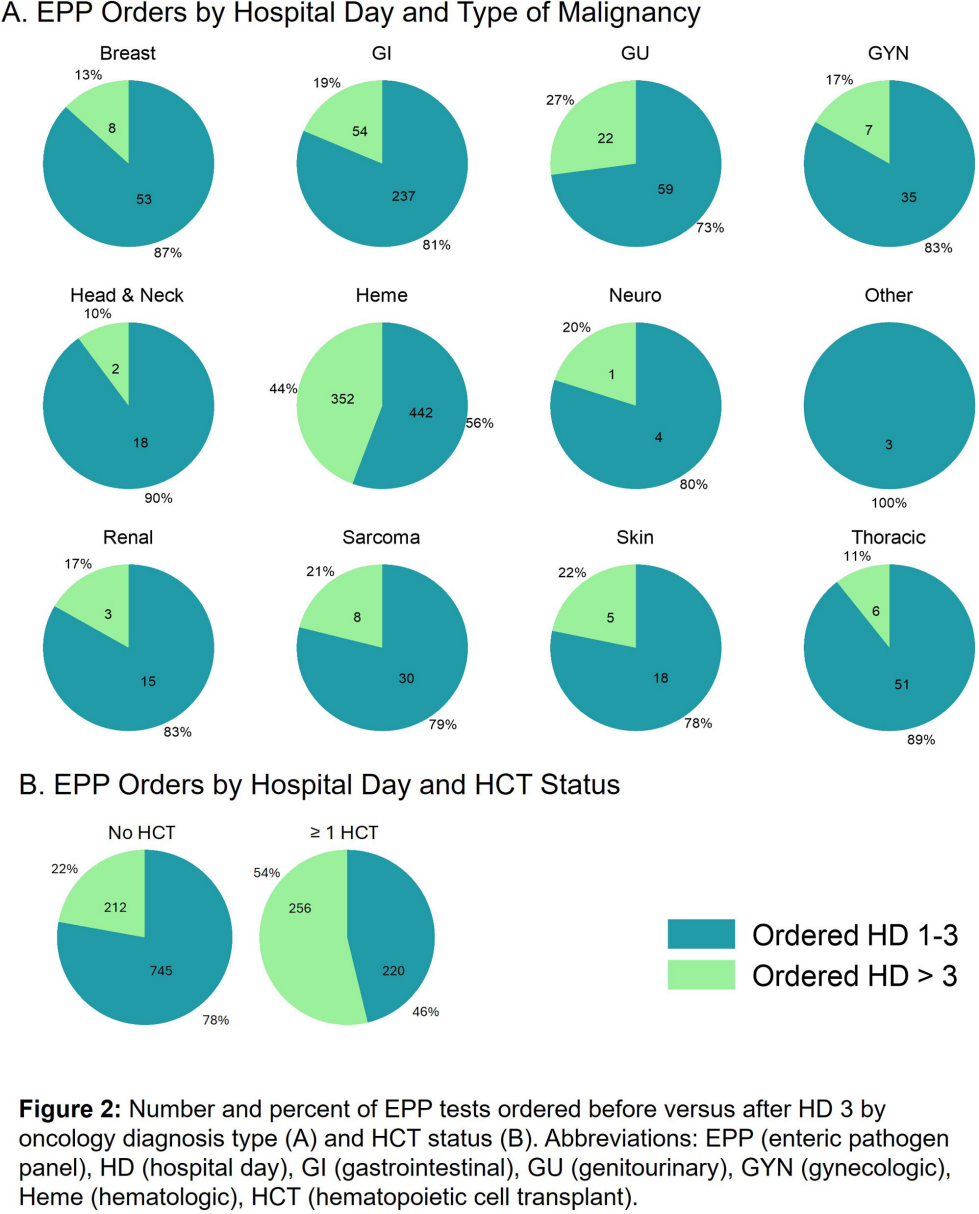

**Figure:**